# Modified Prophylactic Donor Lymphocyte Infusion (DLI) in an Adult T Cell Lymphoma/Leukemia (ATLL) Patient—Modality of Relapse Prevention

**DOI:** 10.3390/diseases12090210

**Published:** 2024-09-11

**Authors:** Alexandra Ionete, Alexandru Bardas, Zsofia Varady, Madalina Vasilica, Orsolya Szegedi, Daniel Coriu

**Affiliations:** 1 Fundeni Clinical Institute, 022328 Bucharest, Romania; varadyzsofia@gmail.com (Z.V.); v.madalina@yahoo.com (M.V.); daniel_coriu@yahoo.com (D.C.); 2 Faculty of General Medicine, University of Medicine and Pharmacy “Carol Davila”, 020021 Bucharest, Romania; orsolya.szegedi96@yahoo.com

**Keywords:** donor lymphocyte infusion (DLI), adult T-cell leukemia/lymphoma (ATLL), allogeneic stem cell transplantation, prophylactic DLI, stem cell transplantation (HSCT), post-transplant cyclophosphamide (PTCy)

## Abstract

Adult T-cell Leukemia/Lymphoma (ATLL) is a rare but aggressive malignancy associated with the human T-cell lymphotropic virus type 1 (HTLV-1). ATLL is a challenging malignancy characterized by its aggressive nature and poor prognosis. Despite advancements in treatment, relapse rates remain high. Donor lymphocyte infusion (DLI) is a promising therapeutic option post-hematopoietic stem cell transplantation (HSCT) to prevent relapse. However, the prophylactic use of DLI in ATLL patients remains underexplored. We report the case of a 45-year-old female diagnosed with ATLL. Following induction chemotherapy and successful HSCT, a modified prophylactic DLI regimen was administered, consisting of gradually increasing doses of donor lymphocytes. The patient demonstrated a favorable response with no significant graft-versus-host disease (GVHD) and maintained remission over a 40-month follow-up period, suggesting a potential benefit of this approach. This case highlights the potential efficacy and safety of modified prophylactic DLI in ATLL patients, warranting further investigation. Our findings suggest that modified prophylactic DLI is a viable option for ATLL patients post-HSCT, offering a balance between efficacy and safety. Future research should focus on optimizing DLI protocols and exploring biomarkers for response prediction.

## 1. Introduction

Adult T cell lymphoma/leukemia (ATLL) is a rare and aggressive T cell neoplasm with general dismal outcomes. In some patients with aggressive ATLL, long survival can be seen post allogeneic stem cell transplantation; although, relapses are common.

ATLL occurrence is related to chronic human T-lymphotropic virus1 (HTLV1) infection, an RNA virus of the family Retroviradae, genus deltaretrovirus. It is mainly encountered in regions with a high HTLV1 prevalence. Such endemic regions are southwestern Japan, sub-Saharan Africa, Latin America, the Caribbean region, the Middle East, and Australia–Melanesia [1]. Worldwide HTLV1 carriers were estimated at 20 million in 1993 and 10 million in 2012 [2].

In Europe, Romania was the only endemic region. In blood donors, the seroprevalence was reported to be 0.64% [3] compared to other European countries, where HTLV1 blood donor seroprevalence was 0.004% [4]. Most cases of ATLL in Europe were immigrant patients from endemic areas or descendants from parents from previously mentioned areas.

The HTLV1 infection in Romania is thought to have been caused by means of a horizontal transmission in the 1990s, similar to the outbreak of HIV-1 [5], and, from a molecular point of view, the viral strains present in Romania belong to the TC subgroup of the cosmopolitan a-genotype [6]. In Romania, in a single-center report by Bumbea et al. [7], 56 patients with aggressive ATLL had a median age of onset of 42.5 years with a median survival of 6.5 months. Patients that achieved a complete response (CR) and underwent allogeneic hematopoietic stem cell transplantations (allo-HSCT) had an OS at 1 year and 2 years after the allo-HSCT of 57.1% and 42.9%, respectively.

In a retrospective data analysis from EBMT Registry, the 3-year OS rate from a cohort of 17 ATLL patients with allo-HSCT was 34.3% [8]. A Romanian study reported a 1-year OS rate of 62.5%, 2-year OS rate of 50%, 3-year OS rate of 37.5%, and a median post-transplant survival for Romanian ATLL-transplanted patients of 19.5 months (range 2.3–44.2 months) [9].

Post-transplant relapse is still a major problem.

Donor lymphocyte infusion (DLI) has a well-established role in the treatment of relapsed disease after allogeneic HSCT. DLI is a form of allogeneic immunotherapy that can induce long-lasting remission by enhancing the graft-versus-tumor/leukemia (GVT/GVL) effect [10,11], an effect due to the infusion of CD3+ T lymphocytes [12,13,14]. After allo-HSCT, tissue damage is gradually repaired. In this process, donor dendritic cells (DCs) replace recipient DCs in the first 6 months post-transplant. Consequently, the donor–patient immune subsets progressively adapt. This explains why higher numbers of T cells can be administered without inducing severe GVHD (<10^5^ CD3+ cells/kgc after 3 months, to 10^6^ CD3+ cells/kgc at 6 months) [15].

As a general rule, DLIs should be administered in the absence of tissue damage and in the absence of inflammatory processes, i.e., in the absence of GVHD and infection [16,17].

Therapeutic DLI has a well-established role in treating relapses of various post-transplant hematological malignancies. Response and survival rates after therapeutic DLI depend on several factors: characteristics and genotype of the underlying disease, size of tumor burden, rate of disease proliferation, donor type, and clinical status of the patient [18,19,20].

In particular, natural killer (NK) cells provide control of leukemic activity. By inducing a tolerance that occurs over time, NK cells lose their anti-leukemic response [21]. Other subsets, such as gamma/delta T cells, have a prolonged anti-leukemic effect [22].

Although therapeutic DLI has been introduced as a treatment option for relapsed disease after allo-HSCT, response rates and long-term survival remain unfavorable [23,24,25].

Both preemptive DLI, for mixed chimerism or molecular/cytogenetic relapse, and prophylactic DLI, for hematological malignancies with increased risk of relapse, have also been studied, with promising results [17,26,27].

There are 2 major types of DLI:

a. Conventional DLI (cDLI) is obtained by leukapheresis of unmobilized peripheral blood.
b. Modified DLI (mDLI) is obtained by leukapheresis of mobilized peripheral blood with GCS-F.

Both cDLI [28,29] and mDLI [30,31] have shown demonstrated efficacy in the prophylaxis and treatment of post allo-HSCT AML relapses. cDLI is used in Western countries, using PT-CY based protocols. mDLI, developed by Peking University, is used by the Chinese, using ATG-based protocols, usually higher doses (between 1 × 10^7^ and 1 × 10^8^ CD3+ cells/kgc for both haploidentical donors and HLA-matched donors) [31,32,33,34,35] than those used in cDLI (between 1 × 10^5^ and 1 × 10^6^ CD3+ cells/kgc for haploidentical donor; between 1 × 10^7^ and 1 × 10^8^ CD3+ cells/kgc for HLA-matched donor) [17,35,36]. In addition, mDLI is followed by brief immunosuppression with cyclosporine A (CsA) or methotrexate (MTX) [37], whereas cDLI is not used with GVHD prophylaxis [12].

Since mobilization with G-CSF changes the cellular composition and cytokine profile of the DLI graft, it is possible that mDLI reduces the incidence of GVHD [37] and has a stronger GVT effect by inducing an immunomodulatory effect [38,39,40].

The time interval between transplantation and DLI administration has an important impact on the morbidity and severity of DLI-associated GVHD [31]: the shorter this interval, the higher the risk of GVHD occurrence.

The amount of CD3+ cells is another important factor for DLI-associated GVHD. An early comparative study between dose-escalated cDLI and single-dose cDLI in relapsed CML after allo-HSCT showed that dose-escalation was associated with a lower incidence of GVHD but with similar results in terms of disease control [41].

Too-early administration of prophylactic/pre-emptive DLI (within the first 40 days) after allo-HSCT may be affected by ATG remaining in the body [42,43].

## 2. Materials and Methods

In this case, we present a 46-year-old female patient, with no personal pathologic history, with no significant hereditary collateral antecedents, diagnosed in August 2020 with ATLL (Adult T cell leukemia/lymphoma), with tumor determinations of the chest wall, breast, and bone (see Table 1).

The patient has an apparent onset of symptoms in June 2020, with the appearance of a tumor formation in the left breast. The August evaluation in the Hematology Clinic indicates the following:

− Clinical: one tumor with 1.4 cm diameter situated in latero-thoracic area, without adenopathy or hepatosplenomegaly.
− CBC (Complete blood count) with mild normochromic normocytic anemia (Hb = 10.1 g/dL).
− Biochemistry within near-normal limits except for slightly increased LDH (413 U/L) (normal value: 81–234 U/L).
− anti-HTLV-1: positive.
− Heart echo: EF (the ejection fraction) 65%, mitral insufficiency grade I, tricuspid insufficiency grade II, mild PTH.
− Biopsy of the tumor formation and immunohistochemistry (IHC): diffuse malignant lymphoid tumor proliferation with banded disposition of large, pleomorphic cells, rounded or incised nucleus, vesicular, nucleolated, pale basophilic cytoplasm; rare tumor cells with high degree of anaplasia are also present. IHC tests: large T-cell tumor proliferation, diffusely positive for CD3, focal positive for CD25, expressing CD30 (activated lymphocyte marker) in isolated cells and negative for CD20 (marker B), negative for EMA (epithelial membrane antigen), negative for ALK (anaplastic lymphoma kinase), negative for Granzyme B, negative for CD56 (NK marker). Due to anti-HTLV1 being positive, the histopathological (HP) classification is adult T-cell malignant lymphoma (ATL).
− Bone marrow biopsy (PBO): very rare, small reactive interstitial lymphoid infiltrates with small, mature cell.
− CT TAP: 2.6/3.8/3 cm mass at the level of the right anterior thoracic wall, causing lysis of the anterior arch of rib II; osteolytic mass with max diam 3/4/3.8 cm involving the lateral arch of rib VIII right; nodular formation 1.2/1.2 cm spontaneously hypodense, hypocaptant, with discrete iodophilic peripheral halo of hepatic segment VI.

Several studies confirm the effectiveness of the antiviral combination of Zidovudine (AZT) and Interferon-α (IFN-α) that has become a standard of care for ATLL patients [43,44,45,46,47,48,49,50]. A UK retrospective study showed the efficacy of AZT and IFN combined with chemotherapy in ATLL patients [50].

The patient receives antiviral treatment with AZT and IFN-α and from 13 August 2020 CHOEP treatment (CFA 1 gr, ETO 150 mg × 3 days, EPI 70 mg, VCR 2 mg), and intrathecal administration of MTX 15 mg, ARA-C 40 mg, and DXM 4 mg is started.

On 4 September 2020, CHOEP II and intrathecal II administration of MTX 15 mg, ARA-C 40 mg, and DXM 4 mg is administered.

PET CT (21 September 2020) (after 2 CHOEP treatments and 2 intrathecal MRX administrations): osteolytic lesions, some with cortical bone disruption and extension into adjacent, metabolically active, soft tissue located at the level of the right C II anterior costal arch (SUV lbm 1.92) and left lateral C VII (SUV lbm 3.24).

Following the PET/CT result, it is decided to maintain the same treatment.

On 30 September 2020, CHOEP III is administered, well tolerated. 

CT TAP 4 October 2020: clear dimensional regression of the osteolytic lesions at the level of costal arches II and VIII on the right side, with remaining tissue component visible only at the level of that of arch VIII. No pulmonary lesions suggestive of secondary determinations. Mildly progressive hepatomegaly. Liver lesion (segment VI) compatible with tumor substrate, dimensional regression compared to the examination of 8 August 2020. No new lesions detectable by CT. No subdiaphragmatic adenomas.

Subsequently continued with the same treatment pattern until CHOEP VI (last on 9 December 2020).

Allo-HSCT is recommended to be conducted early, right after the response to the first line of therapy. The chemotherapy response is not durable, and its continuation leads to high toxicities [51,52]. This is why the patient should be referred to a transplant center as soon as possible [52,53,54,55]. The standard approach is to look for related/unrelated donors from the diagnosis phase [55,56].

PET-CT 7 January 2021 (before allo-HSCT): moderate metabolic activity posterior mediastinal adenopathies recently appeared and reduced metabolic activity of pre-existing bone lesions.

Not having a familial compatible donor (MRD), MUD was sought and found: female unrelated donor, 9/10 compatibility with allelic mismatch B, Rh group A positive CcEekk, CMV negative.

Female recipient with group B Rh positive CcEekk, CMV positive.

Both myeloablative and reduced intensity conditioning (RIC) were used in patients with ATLL. RIC regimens are increasingly used, and prospective sequential studies have revealed the relative safety and promising efficacy of allo-HSCT with RIC [56,57,58,59]. The intensity of conditioning should be determined by the patient’s associated comorbidities at the time of transplantation.

For the case presented, a RIC regimen was used: Melfalan 140 mg total dose/m^2^ and Flu 160 mg total dose/m^2^, GVHD prophylaxis with PTCy + Tacro + MMF.

Day zero was in 22 January 2021: 8.96 × 10^6^ CD34+ cells/kgc were administered (previously cryopreserved due to COVID pandemic). Because of the high amount of stem cells received for her (12.3 × 10^6^ CD34+ cells/kgc), part of the cells was cryopreserved as DLI.

Evolution post allo-HSCT:

1. Minimal nausea, no mucositis.
2. Day +4 fever with negative blood cultures, treated and solved with Meronem + Linezolid + Colistin.
3. Secondary hypertension of tacrolimus on day +8.

No other complications or organ toxicity.

Slow grafting from day +18.

Although allo-HSCT has the potential to cure ATLL, relapse/progression of ATLL after allo-HSCT remains a major obstacle. The prognosis of ATL patients who relapse after allo-HSCT is extremely poor, with a two-year OS of approximately 10%. However, some long-term survivors have been reported following immunosuppression tapering and withdrawal [59,60].

Patients who were able to receive DLI had better survival, suggesting the existence of GvATLL even after relapse [59,60].

Due to partial remission of the disease before the transplant procedure (see PET-CT 7 January 2021) and to the increased risk of relapse, resume antiviral treatment from day +33 only AZT, without IFN, stopping immunosuppression at 5 months post allo-TCSH and administer 3 prophylactic doses of DLI at 10, 11, and 13 months respectively, as follows:

1. 17 November 2021 (approximately 10 months): DLI D1: 0.5 × 10^6^ CD3+ cells/kgc;
2. 29 December 2021 (approximately 11 months): DLI D2: 1 × 10^6^ CD3+ cells/kgc (desired dose)/1.36 × 10^6^ CD3+ cells/kgc (administered dose);
3. 16 February 2022 (approximately 13 months): DLI D3: 5 × 10^6^ CD3+ cells/kgc (desired dose)/5.44 × 10^6^ CD3+ cells/kgc (administered dose). All DLIs were modified DLIs, given prophylactically, patient having 100% donor chimerism, negative PCR-HTLV and negative PET-CT.

22 March 2022 (approximately 1 month after last DLI dose): occurrence of cutaneous GVHD at 56% of surface associated with some lesions in the mouth and mouth ulcers in oral cavity. The used treatment was methylprednisolone 0.8 mg/kgc/day then taper and stop in 3 weeks (on 12 May 2022) with complete resolution of cutaneous GVHD.

## 3. Results

Subsequently, there is an appearance of ocular GVHD with minimal corneal lesions. The patient is a chronic consumer of artificial tears due to corneal dryness; therefore, it is likely that the corneal lesions given by GVHD have become chronic.

PET-CT 11 May 2021 (3 months after allo-TCSH): of complete metabolic response to oncologic treatment.
Chimerism at different time intervals (1, 2, 3, 4, 6, 7.5, 9.5, 15, 17, 22, 25, 34, 40 months) were all 100%.
Determination of Ig (A, G, M) at 6, 18, 25 months: normal values.

The patient is alive, still under our observation, in viral and imagistic remission, with full immune reconstitution and good quality of life at more than 3 years post-transplant.

In Romania, prophylactic DLI is not used yet. Mostly used are therapeutic and preemptive DLI.

In the case presented, mDLI (part of the initial cryopreserved graft) was used, three escalated doses, without additional immunosuppression, in order to prevent a relapse of a disease with high relapse potential.

## 4. Discussion

For this case, we wanted a GVL effect rather than a toxicity of the conditioning. This was the reason for using RIC.

The incidence of ATLL relapse after transplantation is 40%. The therapeutic options in case of relapse for these patients are limited [61,62].

Literature shows that the GVHD effect after DLI is not always accompanied by the GVL effect. Generally, in lymphomas, the occurrence of GVHD after DLI is accompanied by the GVL effect. We consider that in this case, the GVHD effect is accompanied by GVL effect (at present, we are at 30 months after the last dose of DLI, and the patient is in complete remission). We usually stop after taking 3 doses of DLI for prophylactic DLI (whether or not GVHD occurs).

In three cases of ATLL relapse after allo-HSCT, stopping or reducing immunosuppression resulted in conversion at complete remission [62]. This would lead to the idea that the GVL effect in ATLL exists [62,63].

It should be kept in mind that although DLI administration increases the GVL effect and control of the underlying disease, DLI administration is not without adverse effects: GVHD and myelosuppression (mainly neutropenia and thrombocytopenia) [63].

DLI is an allogeneic immunotherapy after allo-TCSH that provides a GVL effect.

This case underscores the potential of modified prophylactic DLI in preventing ATLL relapse. Compared to standard protocols, the gradual escalation of donor lymphocytes may mitigate the risk of GVHD while enhancing anti-leukemic effects. It is important to underline that using PTCy as GVHD prophylaxis offers the possibility to reduce the incidence of acute and chronic GVHD, to stop immunosuppression earlier, and to administer prophylactic DLI with lower risk of GVHD. The literature review indicates limited but supportive evidence for this approach in ATLL, highlighting the need for larger studies to validate these findings. The success of DLI may be attributed to enhanced immune surveillance and targeting of residual malignant cells.

## 5. Conclusions

Our findings suggest that modified prophylactic DLI is a viable option for ATLL patients post-HSCT, offering a balance between efficacy and safety. Future research should focus on optimizing DLI protocols and exploring biomarkers for response prediction.

## Figures and Tables

**Table 1:** Diagnosis, Evolution, and Treatment.

Diagnosis	ATLL
Age	46
Sex	Female
June 2020	Left breast tumor formation
July 2020	Tumor biopsy: non-Hodgkin’s T-cell malignant lymphoma
August 2020	➢ Clinical: 1.4 cm latero-thoracic tumor, without adenopathy or hepatosplenomegaly ➢ CBC: mild normochromic normocytic anemia (Hb = 10.1 g/dL) ➢ Biochemistry: slightly increased LDH (413 U/L) (normal value: 81–234 U/L) ➢ Anti-HTLV1: positive ➢ Heart echo: EF 65%, mitral insufficiency grade I, tricuspid insufficiency grade II, mild PTH ➢ Biopsy of the tumor formation and ex IHC: ATLL ➢ Bone marrow biopsy (PBO): very rare, small reactive interstitial lymphoid infiltrates with small, mature cells. ➢ CT TAP: 2.6/3.8/3 cm mass at the level of the right anterior thoracic wall, causing lysis of the anterior arch of rib II; osteolytic mass with max diam 3/4/3.8 cm involving the lateral arch of rib VIII right; nodular formation 1.2/1.2 cm spontaneously hypodense, hypocaptant, with discrete peripheral halo iodophilic VI hepatic segment.
Treatment	➢ AZT + IFN-α ➢ 6 × CHOEP + intrathecal MTX (13 August 2020–9 December 2020)
Assessment after 2 × CHOEP	PET CT: osteolytic lesions, some with cortical bone disruption and extension into adjacent, metabolically active, soft tissue, located at the level of the right C II anterior costal arch (SUV lbm 1.92) and left lateral C VII (SUV lbm 3.24).
Assessment after 3 × CHOEP	CT TAP: clear dimensional regression of the osteolytic lesions at the level of costal arches II and VIII on the right side, with remaining tissue component visible only at the level of that of arch VIII. No pulmonary lesions suggestive of secondary determinations. Mildly progressive hepatomegaly. Liver lesion (segment VI) compatible with tumor substrate, dimensional regression compared to the first examination (8 August 2020). No new lesions detectable by CT. No subdiaphragmatic adenomas.
Assessment before allo-TCSH	PET CT: metabolically active posterior mediastinal adenopathies, recently appeared. Reduction in the metabolic activity of pre-existing bone lessions.
Allo-HSCT	Recipient: female, 47 kg, B positive CcEekk, CMV positive, VHB negative, VHC negative, EBV postive, HIV negative, HTLV positive, toxoplasma positive Donor: female, mismatched unrelated donor 9/10 alelic B, 56 kg, A positive CcEekk, CMV negative, VHB negative, VHC negative, EBV positive, HIV negative, HYLV negative, toxoplasma positive Conditioning regimen: Melfalan 140 mg total dose/m^2^ and Flu 160 mg total dose/m^2^ GVHD prophylaxis: PTCy + Tacro + MMF. Date: 22 January 2021 PBSC (peripheral blood stem cells) graft administered: 8.96 × 10^6^ CD34+/kgc Slow grafting from day +18
Post allo-HSCT	➢ Day + 33: AZT ➢ 3 months: PET-CT of complete metabolic response to oncologic treatment ➢ 5 months: immunosuppression was stopped ➢ 9 months: PCR HTLV1 negative, PCR HTLV2 negative ➢ Chimerism was always 100% donor ➢ 3 DLI prophylactic doses at 10, 11 and 13 months respectively, as follows: ◦ 10 months: DLI D1: 0.5 × 10^6^ CD3+ cells/kgc ◦ 11 months: DLI D2: 1.36 × 10^6^ CD3+ cells/kgc ◦ 13 months: DLI D3: 5.44 × 10^6^ CD3+ cells/kgc ➢ Approximately 1 month after last DLI dose: occurrence of GVHD cutaneous 56% and mouth ulcers in oral cavity: medrol 0.8 mg/kgc/day then taper and stop in 3 weeks, with complete resolution of cutaneous GVHD. ➢ Subsequently: appearance of ocular GVHD with minimal corneal lesions. ➢ Determination of Ig (A, G, M) at different time intervals: normal values

## Data Availability

The data are accessible from the corresponding author upon reasonable request and taking into account privacy concerns.
